# Clinical Characteristics and Prevalence of Atrial High-Rate Episodes in Patients With Cardiac Implantable Electronic Devices

**DOI:** 10.7759/cureus.75380

**Published:** 2024-12-09

**Authors:** Surachat Jaroonpipatkul, Thipsukhon Sathapanasiri, Chananan Maliang, Apichai Pokawattana, Leenhapong Navaravong

**Affiliations:** 1 Division of Cardiology, Rajavithi Hospital, College of Medicine, Rangsit University, Bangkok, THA; 2 Bangkok Health Research Center, Bangkok Hospital Headquarter, Bangkok Hospital, Bangkok, THA; 3 Department of Cardiology, Utah Valley Hospital, Intermountain Health, Provo, USA

**Keywords:** atrial fibrillation, atrial high-rate episodes, cardiac implantable electronic device (cied), heart arrhythmia, thailand

## Abstract

Background

Atrial high-rate episodes (AHREs) detected by cardiac implantable electronic devices (CIEDs) are indicative of future clinical atrial fibrillation (AF) and stroke risk. This study aimed to investigate the prevalence of AHREs among Thai patients with CIED implantation and identify associated risk factors.

Methods

A retrospective observational study enrolled 278 CIED patients with AHREs lasting five minutes to 24 hours, with an average atrial rate ≥ 175 bpm, excluding known clinical AF at device implantation. Data were collected from electronic and paper medical records, and statistical analyses included Student's t-test, Mann-Whitney U test, and multivariable logistic regression.

Results

Of the 278 patients, 52 with pre-existing AF diagnoses were excluded. The median age was 64.86 years, with a balanced gender distribution. Hypertension was observed in 121 (53.54%) patients and diabetes in 62 (27.43%) patients. Following the designated follow-up period, 58 (20.79%) patients experienced AHREs, and eight (3.53%) developed clinical AF. Multivariable analysis confirmed diabetes as a significant risk factor for AHREs.

Conclusion

This study underscores the importance of monitoring AHREs for early detection of AF and stroke risk in Thai patients with CIEDs. Diabetes emerged as a significant risk factor, highlighting the need for personalized management strategies in clinical practice.

## Introduction

Atrial fibrillation (AF) is well-established as a significant independent risk factor for stroke, contributing markedly to increased morbidity, mortality, and extended hospital stays [1,2]. Clinicians commonly assess stroke risk in AF patients using stratification tools like the CHADS2 (congestive heart failure, hypertension, age ≥ 75 years, diabetes mellitus, stroke) and CHA2DS2-VASc (congestive heart failure, hypertension, age ≥ 75 years, diabetes mellitus, stroke or transient ischemic attack, vascular disease, age 65 to 74 years, sex category) scores, which take into account factors such as age, sex, hypertension, congestive heart failure, diabetes, and myocardial infarction [3]. Despite AF's profound impact on health outcomes and healthcare costs, there remains a critical need for continued research and the development of more personalized treatment strategies.

AF is often preceded by subclinical atrial tachyarrhythmias, including asymptomatic or silent atrial high-rate episodes (AHREs). It is essential that patients with AHREs are evaluated not only for overt AF but also for other stroke risk factors [4,5], highlighting the importance of detecting AHREs.

Cardiac implantable electronic devices (CIEDs), such as pacemakers, implantable cardioverter-defibrillators (ICDs), and cardiac resynchronization therapy (CRT) devices, equipped with atrial leads, have the capability to detect various forms of atrial tachyarrhythmias, such as atrial flutter, atrial tachycardia, and AF. These arrhythmic episodes, frequently asymptomatic, are detected through long-term, continuous rhythm monitoring and are classified as AHREs. These episodes are strongly associated with an elevated risk of progression to clinical AF and thromboembolic events, including stroke. Such episodes are classified as AHREs [6]. It is vital to differentiate AHREs from asymptomatic paroxysmal AF, which is generally diagnosed through surface electrocardiography, as some AHREs might actually be false positives or artifacts rather than true instances of atrial tachyarrhythmias. This distinction is crucial for accurate diagnosis and appropriate management. The criteria for defining AHREs can differ between studies; however, a commonly accepted threshold is an atrial rate of ≥ 175 beats per minute (bpm), which is associated with a significant increase in the risk (approximately three to five times higher) of developing clinical AF [7]. Nonetheless, the majority of studies further refine the definition of AHREs by specifying that these episodes must last for more than five to six minutes to be clinically relevant [6]. This duration-based definition is often used to ensure that brief transient arrhythmias are not mistakenly classified as AHREs, thereby improving the accuracy of AF risk assessment.

Various risk factors, including advanced age, hypertension, left atrial enlargement, congestive heart failure, right ventricular apical pacing, and sinus node dysfunction (as an indication for pacing), have been implicated in the development of AHREs, though definitive evidence remains lacking [8,9].

Most AHRE studies have been conducted in Western populations, where AF prevalence and associated risk factors differ significantly. Asian populations, including Thailand, generally report lower AF prevalence, potentially due to genetic or epidemiological factors. Our study aims to address this underrepresentation by providing insights into AHRE prevalence and risk factors in the Thai population, with implications for regional healthcare strategies.

## Materials and methods

Data source and study design

This retrospective observational cohort study involved the extraction of data from the Rajavithi Hospital database, which includes both individual and anonymized information. The dataset comprised demographic details, records of outpatient drug prescriptions, and inpatient care data such as admission dates, length of stay, discharge diagnoses coded according to the International Classification of Diseases, 10th Revision (ICD-10), procedures conducted during hospital stays, and long-term disease outcomes. The study protocol (Protocol number: 64210) received approval from the local IRB/Ethics Committee, ensuring compliance with national regulations and adherence to the principles outlined in the Declaration of Helsinki.

Study populations

This study was conducted at Rajavithi Hospital in Bangkok, Thailand, from January 2015 to May 2020. This study included patients with pacemakers, ICDs, and CRT devices with atrial leads capable of detecting AHREs implanted during this period. Patients included in the study experienced AHREs lasting between five minutes to 24 hours, with an average atrial rate of ≥ 175 beats per minute (bpm). Patients with known clinical AF at the time of device implantation were excluded from the study. The exposure duration, defined as the time from CIED implantation to the last follow-up or occurrence of AHRE, was recorded for each patient.

Outcomes and follow-up

The primary outcome was the prevalence of AHREs among the study population. Secondary outcomes included the identification of risk factors associated with AHREs. The exposure duration varied among patients, reflecting the individualized follow-up periods.

Statistical analyses

Categorical variables are presented as frequencies and percentages, and comparisons were made using the chi-squared test. Quantitative data with skewed distributions are reported as medians with interquartile ranges (IQR) and were compared using the Mann-Whitney U test. Normally distributed data are presented as means with standard deviations (SD) and were compared using the Student’s t-test. For the primary analysis, logistic regression was employed to identify and adjust for risk factors, with results expressed as odds ratios (OR). Statistical analyses were performed using Stata version 17 (StataCorp LLC, College Station, TX). A P-value of < 0.05 for the two-tailed tests was considered statistically significant.

## Results

In Table 1, we summarize the demographic characteristics and clinical profile of the study population. Out of the initial cohort of 278 patients, 52 individuals with pre-existing diagnoses of AF were excluded from the study, resulting in a final study population comprising a diverse demographic profile. Among these patients, the distribution between genders was fairly balanced, with 112 (49.56%) patients being male and 114 (50.44%) female. The median age of the study population was 64.86 years, with a standard deviation of 15.26 years. Throughout the study period, patients were monitored for a median duration of 13 months, ranging from 2.71 to 27.41 months.

**Table 1:** Demographic and clinical characteristics of the patients. The values are presented as number (%) and mean ± standard deviation. * P-value < 0.05 was considered statistically significant.

Baseline characteristics	Total (n = 226)	No atrial high-rate episodes (n = 179)	Atrial high-rate episodes (n = 47)	p-value
BMI (mean ± standard deviation)	23.91 ± 4.17	23.89 ± 4.16	23.97 ± 4.26	0.900
Indication for cardiac implantable electronic devices				
Sick sinus syndrome (n, %)	85 (37.61%)	59 (32.96%)	26 (55.32%)	0.007*
Complete heart block (n, %)	90 (39.82%)	80 (44.69%)	10 (21.28%)	0.004*
Dilated cardiomyopathy (n, %)	26 (11.50%)	20 (11.17%)	6 (12.77%)	0.798
Ventricular tachycardia (n, %)	23 (10.18%)	16 (8.94%)	7 (14.89%)	0.276
Ventricular fibrillation (n, %)	5 (2.21%)	5 (2.79%)	0 (0%)	0.586
Brugada syndrome (n, %)	3 (1.33%)	3 (1.68%)	0 (0%)	1.000
Sudden cardiac death (n, %)	2 (0.88%)	2 (1.12%)	0 (0%)	1.000
Hypertrophic cardiomyopathy (n, %)	2 (0.88%)	2 (1.12%)	0 (0%)	1.000
Procedure				
Pacemaker (n, %)	172 (76.11%)	136 (75.98%)	36 (76.11%)	0.995
Automatic implantable cardioverter-defibrillator (n, %)	34 (15.04%)	27 (15.08%)	7 (14.89%)	-
Cardiac resynchronization therapy (n, %)	20 (8.85%)	16 (8.94%)	4 (8.51%)	-
Gender				
Male (n, %)	112 (49.56%)	93 (51.96%)	19 (40.43%)	0.190
Female (n, %)	114 (50.44%)	86 (48.04%)	28 (59.57%)	-
Age (mean ± standard deviation)	64.86 ± 15.26	64.45 ± 15.47	66.42 ± 14.51	0.430
<65 (n, %)	101 (44.69%)	84 (46.93%)	17 (36.17%)	-
65-74 (n, %)	49 (21.68%)	34 (18.99%)	15 (31.91%)	-
≥75 (n, %)	76 (33.63%)	61 (34.08%)	15 (31.91%)	-
Underlying disease				
Hypertension (n, %)	121 (53.54%)	91 (50.84%)	30 (63.83%)	0.139
Diabetes mellitus (n, %)	62 (27.43%)	48 (26.82%)	14 (29.79%)	0.715
Stroke/transient ischemic attack/thromboembolism (n, %)	18 (7.96%)	12 (6.70%)	6 (12.77%)	0.222
Vascular disease (n, %)	40 (17.70%)	31 (17.32%)	9 (19.15%)	0.830
History of congestive heart failure (n, %)	51 (22.57%)	40 (22.35%)	11 (23.40%)	0.847
Left ventricular ejection fraction <40% (n, %)	39 (17.26%)	30 (16.76%)	9 (19.15%)	0.670
Chronic obstructive pulmonary disease (n, %)	4 (1.77%)	2 (1.12%)	2 (4.26%)	0.192
Current medication and personal history				
Beta-blocker (n, %)	66 (29.20%)	44 (24.58%)	22 (46.81%)	0.004*
Smoking (n, %)	48 (21.24%)	38 (21.23%)	10 (21.28%)	1.000
Alcohol drinking (n, %)	44 (19.47%)	37 (20.67%)	7 (14.89%)	0.417

The prevalence of hypertension was notably high, affecting 121 patients (53.54%) of the study population, while other prevalent comorbidities included 62 (27.43%) patients with diabetes, 18 (7.96%) patients with a history of stroke, and 40 (17.70%) patients with vascular diseases. Additionally, 51 (22.7%) patients had a history of congestive heart failure, and 39 (17.26%) patients exhibited left ventricular ejection fraction (LVEF) values below 40%.

Pacemakers emerged as the predominant type of CIED, accounting for 172 (76.11%) patients in the study population. Following the designated follow-up period, 47 (20.79%) patients of the study cohort experienced AHREs, while clinical AF developed in eight (3.53%) patients. These findings underscore the complexity of the study population and provide insights into the prevalence of comorbidities and cardiac diagnoses among patients with CIEDs.

Upon conducting multivariable analysis, notable associations between AHREs and diabetes were confirmed, highlighting the significance of this comorbidity in predicting AHRE occurrence. These findings are summarized in Tables 2, 3.

**Table 2:** Univariable logistic regression for atrial high-rate episodes. * P-value < 0.05 was considered statistically significant. uOR: univariable odds ratio.

Factors	uOR	95% confidence interval	p-value*
Female	3.17	0.99-10.17	0.052
Body mass index (BMI)	1.00	0.89-1.13	0.956
Age (years)			
<65	Reference	Reference	Reference
65-74	1.71	0.44-6.66	0.442
≥75	1.95	0.59-6.39	0.272
Hypertension	2	0.67-5.96	0.213
Diabetes	2.89	1.03-8.07	0.043*
Previous stroke, transient ischemic attack	1.73	0.88-3.42	0.114
Vascular disease	1.61	0.49-5.28	0.431
Congestive heart failure	1.15	0.36-3.75	0.809

**Table 3:** Multivariable logistic regression for atrial high-rate episodes. * P-value < 0.05 was considered statistically significant. ^+^ Adjusted factors by body mass index (BMI), age, hypertension, previous stroke, vascular disease, and congestive heart failure. mOR: multivariable odds ratio.

Factors	mOR^+^	95% confidence interval	p-value*
Female	3.19	1.00-10.32	0.052
Diabetes mellitus	2.91	1.03-8.22	0.044*

## Discussion

Modern CIEDs incorporate automated algorithms to alert healthcare providers about AHREs. These episodes are increasingly recognized as precursors to clinical AF and stroke. Interestingly, the stroke risk associated with AHREs is notably lower than that observed in patients with ECG-detected AF, likely attributed to the infrequent and brief nature of AHREs [10].

The incidence of AHREs varies widely, ranging from 10% to 70%, influenced by differing definitions and follow-up durations. Utilizing nominal settings with an atrial rate > 175 bpm, chosen based on prior studies demonstrating > 95% sensitivity and specificity for detecting atrial tachycardia/AF episodes [11], our analysis of Thai patients with CIEDs revealed that 20.79% experienced AHREs.

In our study, the incidence of AHREs in the Thai population was found to be slightly lower than that reported in studies conducted in clinical sites across North America, Canada, Australia, and New Zealand, where incidences of 27.6% and 30.4% were observed [12,13]. This aligns with previous research by Banerjee et al., which reported a 21% AHRE incidence in other Asian populations [14]. These differences may be attributed to the varying prevalence of AF among different ethnicities. While AF is more prevalent in cohorts from North America, Europe, and Oceania, its prevalence in Asian populations tends to be lower and less consistent across studies [15].

Our study identifies diabetes as a significant risk factor for AHREs, consistent with prior research suggesting that chronic hyperglycemia, glucose variability, and associated metabolic derangements contribute to atrial remodeling, fibrosis, and arrhythmogenesis [16]. Chronic hyperglycemia, along with blood glucose fluctuations and long-term variability, is associated with atrial fibrosis, oxidative stress, and susceptibility to AF [17]. Notably, diabetes was confirmed as a risk factor for AHREs in our analysis. These findings underscore the need to consider diabetes as a critical factor in risk stratification for AF, particularly in patients with CIEDs. Given the established association between diabetes and thromboembolic risk, these patients may benefit from more intensive monitoring for AHREs and earlier consideration of anticoagulation therapy where clinically appropriate.

These findings could have significant implications for clinical guidelines, highlighting the importance of incorporating diabetes management into AF prevention strategies, particularly for patients with AHREs. Diabetes-specific interventions, such as the use of sodium-glucose cotransporter-2 (SGLT2) inhibitors or glucagon-like peptide 1 (GLP-1) receptor agonists with demonstrated cardiovascular benefits, may offer new opportunities for integrated care in this high-risk population. Future studies are warranted to evaluate the effectiveness of such interventions in mitigating AF risk in patients with diabetes.

The limited availability of remote monitoring systems in Thailand presents a challenge to the optimal management of patients with CIEDs. Remote monitoring is pivotal in the timely detection of AHREs and the subsequent management of associated risks [18]. However, due to infrastructure limitations and uneven access across various regions, not all patients benefit equally from these monitoring techniques.

Identifying risk factors such as diabetes allows for better triaging of patients who may require closer follow-up. Such stratification can be instrumental in the early initiation of anticoagulation therapies, thereby minimizing the risk of thromboembolic events. As highlighted by our findings, personalized management strategies are crucial in regions where technological advancements are yet to be fully integrated into routine clinical practice.

It is essential for future research to explore the implementation barriers and develop strategies that enhance the availability and utilization of remote monitoring in Thailand. This would ensure that all patients, regardless of their geographical location, receive the best possible care in the management of cardiac arrhythmias.

Limitations

This study has several limitations that should be considered when interpreting the results. First, the retrospective observational design may introduce selection bias, as data were collected from existing medical records, potentially affecting the accuracy and completeness of the information. Second, the cohort was limited to a single center in Thailand, with a small sample size, which may limit the generalizability and accuracy of the findings to broader populations or different healthcare settings. Third, the study excluded patients with known clinical AF at device implantation, which might underestimate the prevalence of AHREs among patients with pre-existing conditions. Additionally, the lack of remote monitoring systems and limited access to continuous rhythm monitoring in some regions may have affected AHRE detection rates in our study. In Thailand, the underutilization of remote monitoring likely contributed to the underdetection of AHREs compared to regions where such systems are widely implemented. While cost is often suggested as a barrier, there is no clear evidence to confirm this, and no cost-effectiveness analyses of remote monitoring have been conducted. Addressing this knowledge gap through future research could help guide resource allocation and improve access to remote monitoring technologies. Furthermore, integrating continuous rhythm monitoring and extending follow-up periods in future multicenter studies could provide a more comprehensive understanding of AHRE prevalence and associated risk factors in underrepresented populations. Lastly, the relatively short follow-up period and the variability in follow-up duration across patients may impact the ability to capture longer-term outcomes, such as progression to clinical AF or stroke.

## Conclusions

In conclusion, our study highlights the prevalence of AHREs among Thai patients with CIEDs and identifies diabetes as a significant risk factor for AHRE occurrence. These findings emphasize the critical role of AHRE monitoring in the early detection of AF and stroke risk, offering valuable insights for developing personalized management strategies in clinical practice. While the observed association between diabetes and AHREs aligns with prior research, further validation is essential. Prospective or multicenter studies with larger, more diverse populations and standardized AHRE detection protocols are needed to confirm these findings. Additionally, future research should address potential biases, such as variability in follow-up duration and the underutilization of remote monitoring, to enhance the generalizability and impact of these results.
